# Supplementary material for: Radial bound states in the continuum for polarization-invariant nanophotonics
Source: Nat Commun. 2022 Aug 25;13:4992. doi: 10.1038/s41467-022-32697-z (PMC9411165; doi:10.1038/s41467-022-32697-z)
Supplement: Supplementary file 1 — Supplementary Information [file 41467_2022_32697_MOESM1_ESM.pdf]

Supplementary Information

Radial bound states in the continuum for polarization-invariant nanophotonics

Lucca Kühner[1,2], Luca Sortino[1,2], Rodrigo Berté[1,2,3], Juan Wang[1,2], Haoran Ren[4], Stefan A. Maier[4,5,1,2], Yuri Kivshar[6] and Andreas Tittl[1,2,*]

[1]Chair in Hybrid Nanosystems, Nanoinstitute Munich, Faculty of Physics, Ludwig-Maximilians-Universität München, Königinstrasse 10, 80539 Munich, Germany

[2]Center for NanoScience (CeNS), Faculty of Physics, Ludwig-Maximilians-Universität München, Schellingstrasse 4, 80799 Munich, Germany

[3]Instituto de Física, Universidade Federal de Goiás, 74001-970 Goiânia-GO, Brazil

[4]School of Physics and Astronomy, Monash University, Clayton, Victoria 3800, Australia

[5]The Blackett Laboratory, Department of Physics, Imperial College London, London, SW7 2AZ, United Kingdom

[6]Nonlinear Physics Centre, Research School of Physics Australian National University, Canberra ACT 2601, Australia

*e-mail: andreas.tittl@physik.uni-muenchen.de

Supplementary note 1: Comparison of quality factors in different qBIC systems

There are numerous approaches for realizing quasi bound states in the continuum (qBICs) in photonic metasurfaces, which are all accompanied by certain benefits and drawbacks. We distinguish three classes here for simplicity: accidental and photonic-crystal-based qBICs, qBICs emerging from strongly coupled photonic modes in individual subwavelength resonators, and symmetry-broken qBICs. So far, photonic-crystal-based approaches have shown the highest quality factors, with specific examples reaching around 10^6 [1]. However, they often rely on grating-based approaches and lack flexible tunability of the resonances. Quasi-BICs emerging from strongly coupled modes possess the lowest footprints since the do not rely on array-based approaches [2]. Nevertheless, these resonators have so far been limited to experimental Q factors below 200, often require complex optical excitation with structured light, and exhibit near-fields that are mostly located inside the structures, reducing the spatial overlap with analytes and thus the potential sensing performance.

In contrast, symmetry-broken BICs show exceptional resonance tunability and sustain high-Q resonances down to small array sizes, with record Q-factor values of roughly 18500 [3]. For better comparison, we show some representative works in the field of BIC-based nanophotonics in Table 1. Notably, the radial BIC exhibits the smallest footprint and thus highest Q factor vs. footprint ratio apart from the single disk BIC, which we mentioned above but exclude here due to the complex optical excitation conditions and unsuitable near-field distributions for sensing applications. [4,5] In fact, comparing the same number of unit cells with Ref. [3], the radial BIC exhibits the same quality factor although confined to a much smaller footprint and sustains resonances in the visible where material losses and fabrication imperfections – in contrast to the NIR region – pose significant challenges. In fact, we carried out simulations for our radial BIC geometry, which demonstrate that the ring arrangement of unit cells is favored compared to the arrangement in a 2D array (see Figure S3). We attribute this effect to the improved inter-resonator coupling.

	Ref. 5	Ref. 3	Ref. 4	Ref. 1	Ref. 2	Our work
BIC mechanism	Symmetry-broken	Symmetry-broken	Accidental	Photonic crystal	Strong mode coupling	Symmetry-broken
Excitation	Linear pol.	Lin. Pol.	Lin. Pol.	Lin. Pol	Structured	Lin. Pol.
Resonance wavelength	**VIS**, 750-860 nm	**NIR**, 1490 – 1550 nm	**VIS/NIR,** 825 – 842 nm	**VIS**, 570-850 nm	**IR**, 1500-1600 nm	**VIS**, 700-850 nm
Quality (Q) factor	144	500 - 18511	2750	10^6	Up to 200	100 - 500
Footprint	40000 µm^2	10.6 – 308* µm^2	24000 µm^2	10^8 µm^2 *	0.62 µm^2	2.7 µm^2
Q / footprint	0.0036	66 - 117	0.11	0.01	322	186

*Marked fields were not explicitly given in the manuscript and were estimated from images or spectra.

Supplementary note 2: Tradeoff between electric near-field and resonance modulation

For a radial BIC geometry composed of a lossless dielectric, we expect higher near-fields for decreasing asymmetries, with a maximum value for an infinitesimal deviation from the symmetric case (see, e.g., Ref. [7]), and thus the near-field intensity should peak for the smallest possible value of ΔL, which is still larger than zero. To illustrate this behavior, we added a plot showing the associated near fields for different rings down to ΔL = 5nm in Figure S5.

As is obvious from the plots, the near fields increase tremendously for reduced asymmetries, but enter a saturation regime when going below an asymmetry of ΔL = 25 nm. As a result, a further reduction of the asymmetry will not be accompanied by significant increase of the near fields and thus, the chosen asymmetry is already in the optimum region.

At the same time, in real experiments, there is competition between radiative and parasitic losses, such as material losses, losses induced by surface or edge roughness or even losses related to the statistical variation of the geometrical cross sections of the constituent resonators within the unit cell [8]. These parasitic losses fundamentally limit the achievable quality factors but also the maximum achievable resonance modulations and will consequently diminish resonance modulations for asymmetries below 25 nm.

Our reason to choose a 25 nm asymmetry in experiments was to balance high near field enhancements with sufficient resonance modulation at the experimental condition nonzero losses such that the resonance could still be retrieved reliably at our white light confocal microscope. Taken together, the minimum asymmetry which we used was the optimum value for a finite but not infinitesimally small value of ΔL, see a similar case explained in the connection with for the nonlinear effects (see Ref. [9]).

Supplementary figures

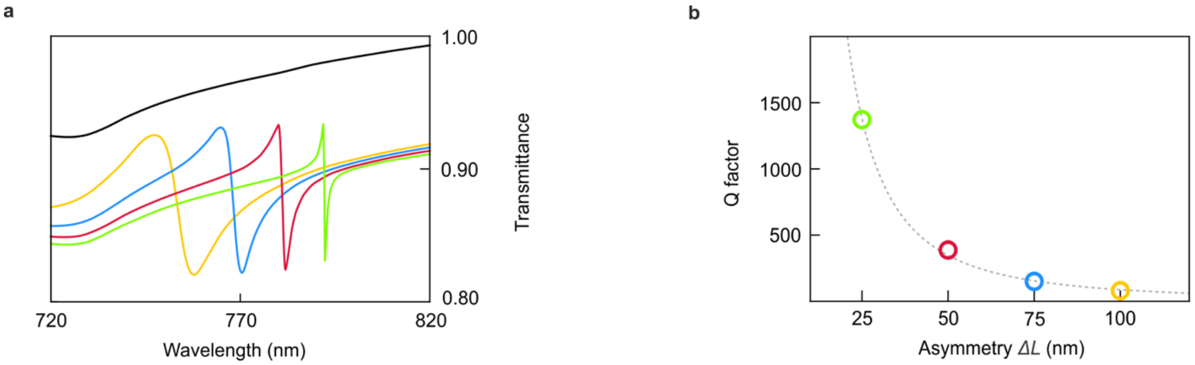

Fig. S1 | Simulated spectral radial BIC responses for different asymmetries. a, Simulated transmittance spectra of radial BIC structures with different asymmetries with *R* = 1.5 µm, where the black response (shifted for clarity) corresponds to the symmetric case. **b**, Extracted Q factors for the spectra shown in (**a**) follow the well-known $(\Delta L)^{-2}$ behavior (fitted as gray dashed line).

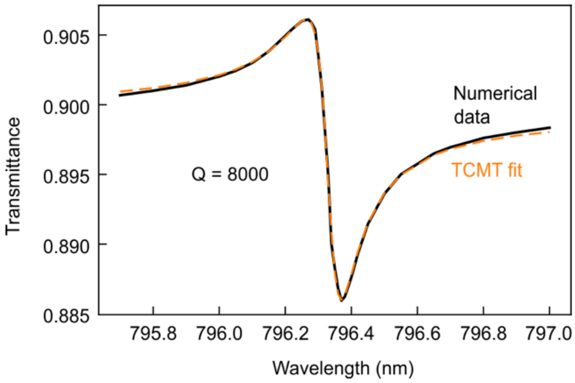

Fig. S2 | Towards the highest Q factors of the radial BIC platform. Numerical data for an asymmetry of *ΔL* = 5 nm and a ring radius of *R* = 1.5 µm show Q factors above 8000.

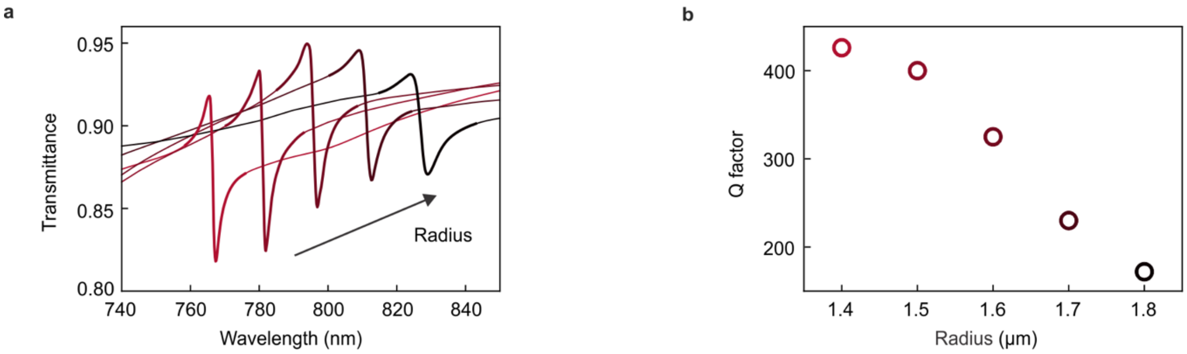

Fig. S3 | Simulated spectral radial BIC responses for different radii. a, Simulated transmittance spectra of radial BIC structures with different radii ranging from *R* = 1.4 µm to *R* = 1.8 µm for *ΔL* = 50 nm. **b**, Extracted Q factors for the spectra shown in (**a**), showing the highest Q factor for the smallest radius.

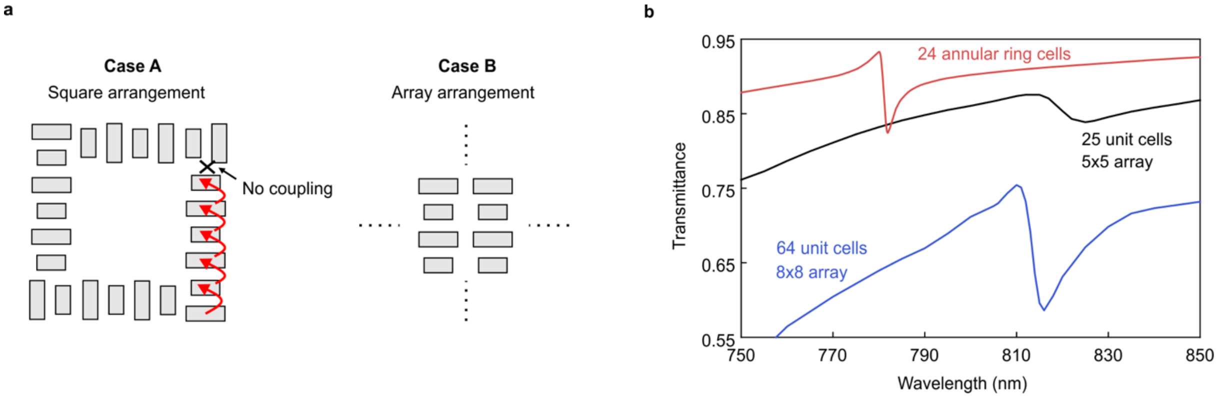

Fig. S4 | Advantages of an annular arrangement of unit cells. a, Different possible arrangements for polarization invariant response (case A) that lacks mode coupling at the edges of the structure and reduced footprints (case B) without polarization invariance and worse performance (see panel b). **b,** Numerical investigation of the difference between resonator arrays of 8x8 (blue curve) and 5x5 (black curve) unit cells arranged in a two-dimensional array compared to 24 unit cells (red curve) arranged in a ring fashion. Clearly, the resonance is sharpest for the ring arrangement.

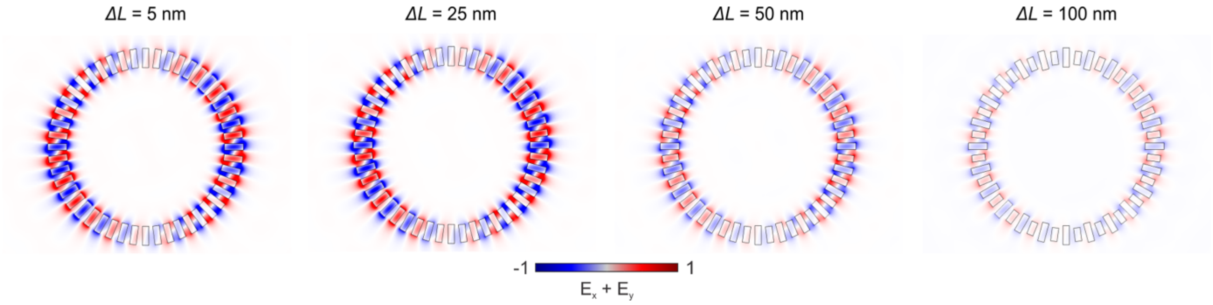

Fig. S5 | Electric near fields for different ring asymmetries. Numerical simulations of the electrical near fields for rings starting with ΔL = 100 nm (right hand side) down to ΔL = 5 nm. The electric near-fields almost saturate below ΔL = 25 nm showing only slight differences between the smallest asymmetries. All near fields are plotted on the same scale.

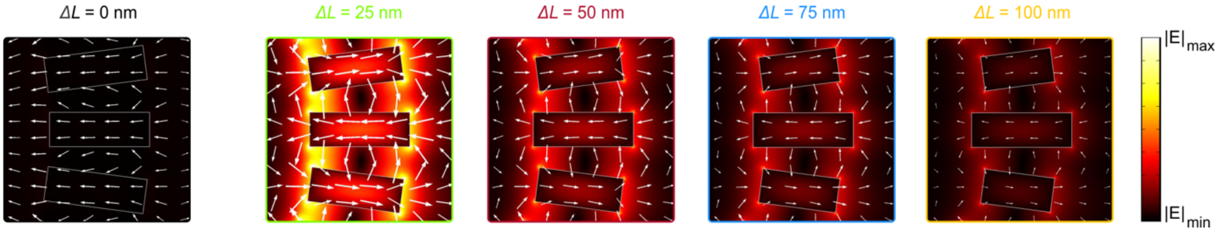

Fig. S6 | Normalized near-field intensities for different asymmetries. Numerical simulations of the near-field intensity for the asymmetries given in Fig. 2 in the manuscript. For better comparison, the near-field intensities are all normalized to the same value.

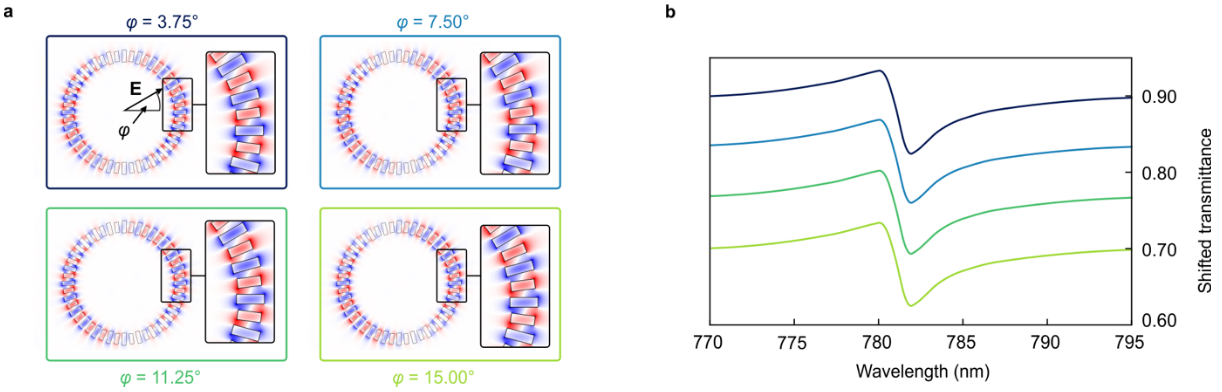

Fig. S7 | Influence of the polarization angle on radial BIC mode formation. a, Electric near fields for different linear polarizations φ of the incident light showing no differences in the mode formation. **b**, Corresponding numerical spectra for the different polarization angles (shifted for clarity) exhibit the same resonance position, Q factor, and resonance modulation.

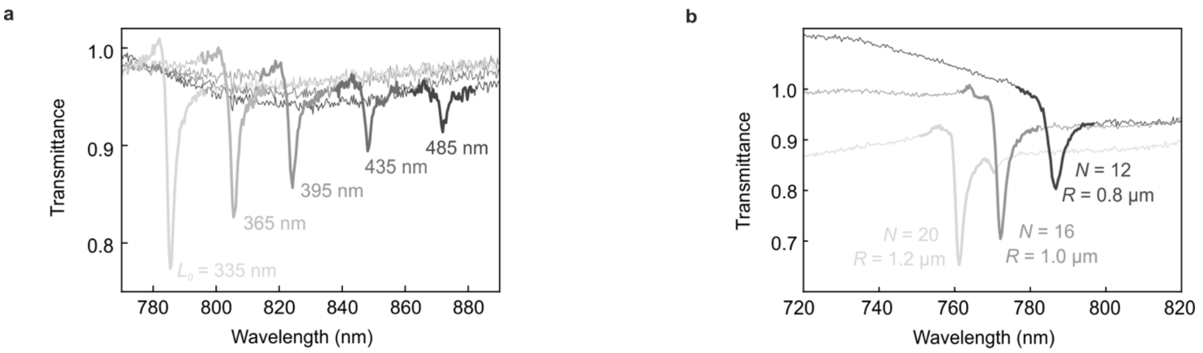

Fig. S8 | Influence of base rod length L_0 and number of unit cells on the optical response of radial BICs. a, Measured transmittance spectra for increasing base rod length L_0 for an asymmetry of ΔL = 50 nm. A red-shift of the radial BIC resonance is observed for higher base length. **b**, White light transmittance spectra for different number of unit cells with ΔL = 50 nm. The Q factor decreases for fewer unit cells due to decreased inter-rod coupling.

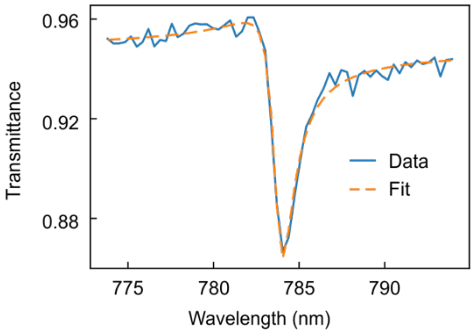

Fig. S9 | Exemplary fit of measured radial BIC transmittance spectra with the TCMT model. A high quality factor above 500 in the visible spectral region was determined for a symmetry-broken ring structure with ΔL = 25 nm and R = 1.4 µm.

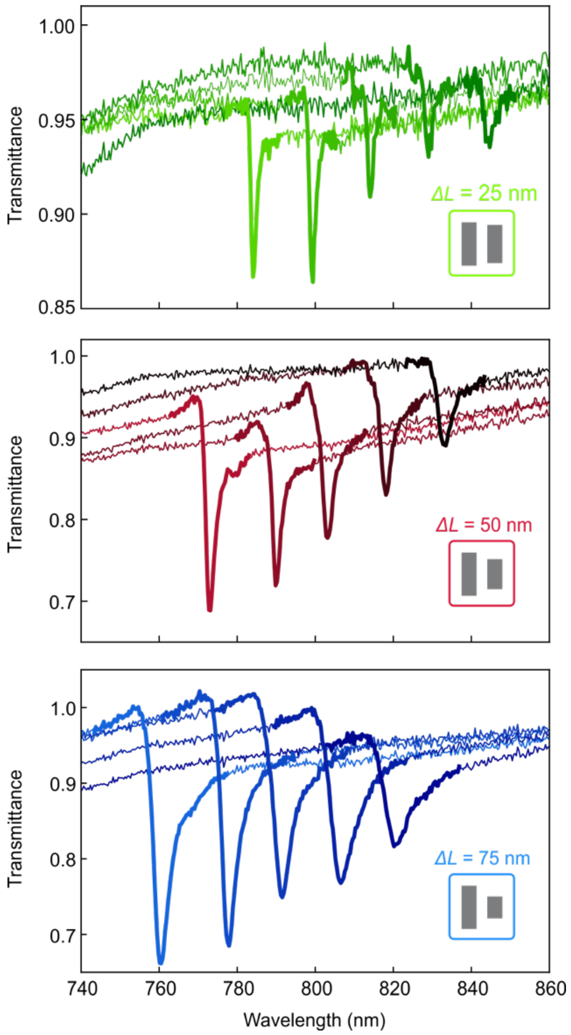

Fig. S10 | White-light transmittance spectra for different asymmetries. Optical transmittance spectra of the radial BIC geometry with different asymmetries for a ring radius of R = 1.5 µm. For the smallest asymmetries, we observe the highest Q factors exceeding 500.

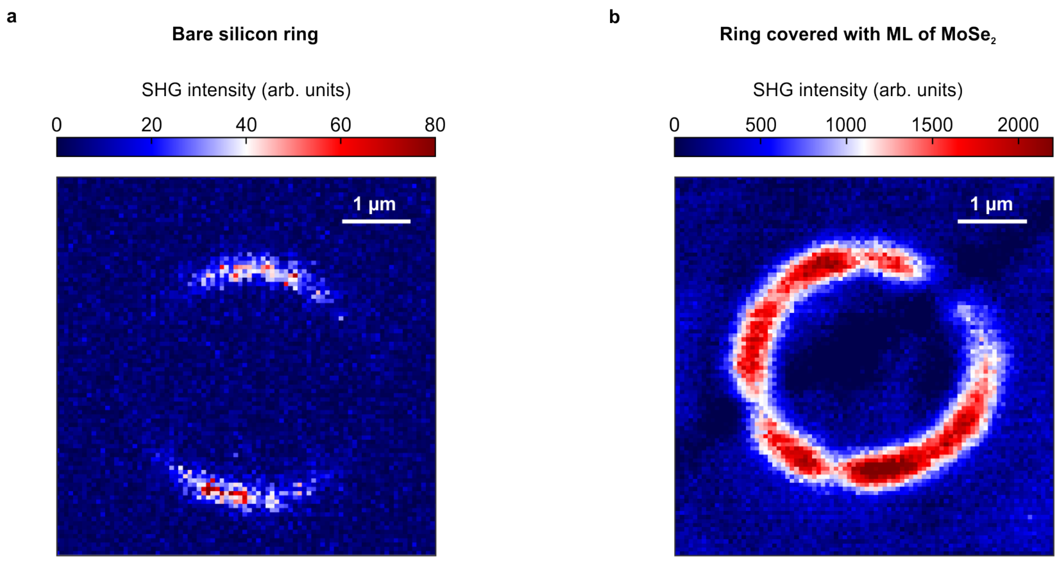

Fig. S11| SHG map comparison of a bare silicon radial BIC structure and a $MoSe_2$ covered ring. Comparison of SHG maps extracted from a bare asymmetric silicon radial BIC geometry in (**a**) and from the same ring covered with a monolayer of $MoSe_2$ in (**b**) plotted on different scales. For comparability of both maps, the background signal is set to zero. The maximum signal obtained from bare silicon structures is more than two orders of magnitude smaller on the whole ring compared to the $MoSe_2$ covered ring and can thus be neglected.

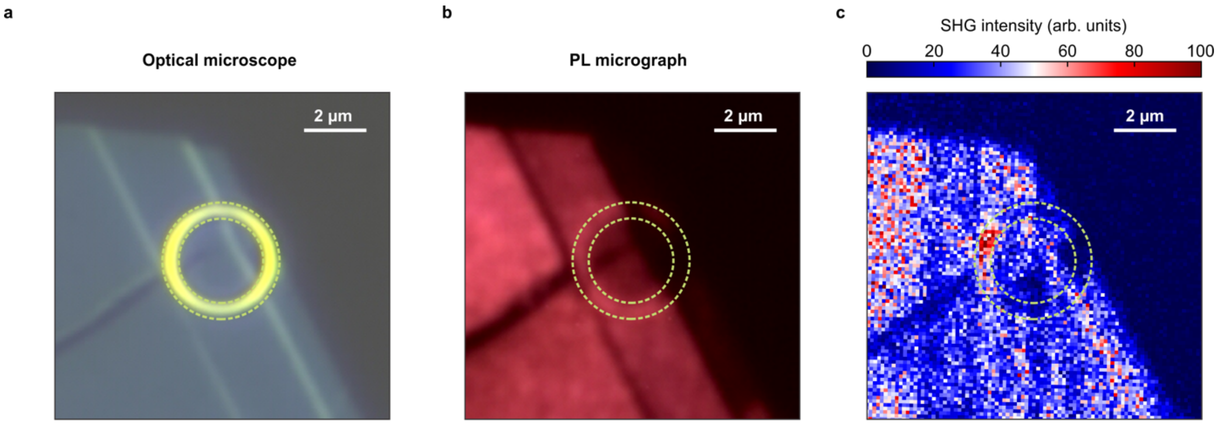

Fig. S12 | SHG map of a symmetric ring covered with a monolayer of $MoSe_2$. a, b, Optical and photoluminescence micrograph of the radial BIC ring which is used for SHG reference maps for the purely symmetric structure. The outline of the ring is indicated as yellow dashed circles for clarity. **c,** SHG map for the symmetric structure. Except for regions where the monolayer is folded, we observe no enhancement of the SHG signal present supporting the BIC-induced SHG enhancement.

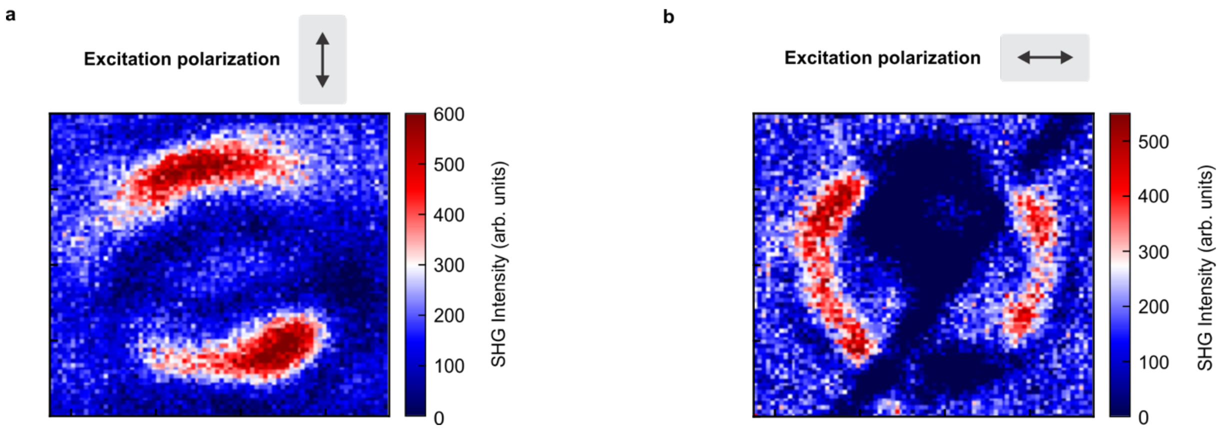

Fig. S13 | Excitation polarization dependent SHG map of the same ring covered with a monolayer of $MoSe_2$. a, Background-corrected SHG map for vertical excitation polarization. **b,** Background-corrected SHG map for horizontal excitation polarization.

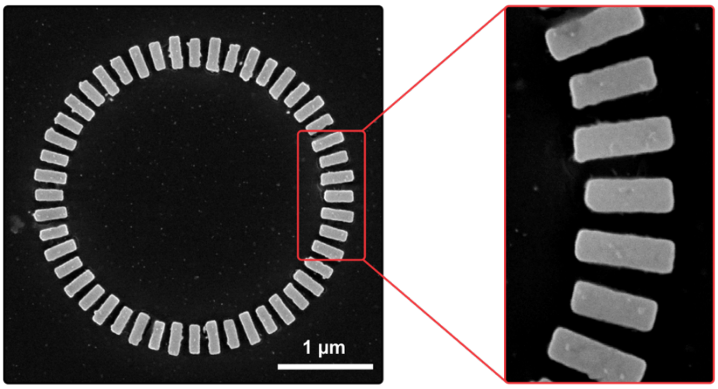

Fig. S14 | Scanning electron micrographs of the fabricated structures.

References

[1] Hsu, C. W. *et al.* Observation of trapped light within the radiation continuum. *Nature* **499**, 188–191 (2013).

[2] Koshelev, K. *et al.* Subwavelength dielectric resonators for nonlinear nanophotonics. *Science* **367**, 288–292 (2020).

[3] Liu, Z. *et al.* High-Q Quasibound States in the Continuum for Nonlinear Metasurfaces. *Phys. Rev. Lett.* **123**, 253901 (2019).

[4] Ha, S. T. *et al.* Directional lasing in resonant semiconductor nanoantenna arrays. *Nat. Nanotechnol.* **13**, 1042–1047 (2018).

[5] Yesilkoy, F. *et al.* Ultrasensitive hyperspectral imaging and biodetection enabled by dielectric metasurfaces. *Nat. Photonics* **13**, 390–396 (2019).

[6] Kang, L. *et al.* Efficient second-harmonic generation in high Q-factor asymmetric lithium niobate metasurfaces. *Opt. Lett.* **46**, 633 (2021)

[7] Koshelev, K. *et al.* Dielectric resonant metaphotonics. *ACS Photonics* **8**, 402-412 (2021)

[8] Kühne, J. *et al.* Fabrication robustness in BIC metasurfaces. *Nanophotonics* **10**, 4305-4312 (2021)

[9] Koshelev, K. *et al.* Nonlinear metasurfaces governed by bound states in the continuum. *ACS Photonics* **6**, 1639-1644 (2019).
